# Race Against Time: How the Autonomy Needs of Adult Rowers of Different Ages Are Met and the Impact of Coaching

**DOI:** 10.1002/ejsc.70065

**Published:** 2025-10-08

**Authors:** David Houchin, Jessica Scott, Robert J. Booth

**Affiliations:** ^1^ Faculty of Education, Health and Wellbeing School of Psychology University of Wolverhampton Wolverhampton UK; ^2^ Centre for Teaching and Learning University of Cambridge Cambridge UK; ^3^ Department of Behavioural Science and Health University College London London UK

**Keywords:** autonomy frustration, autonomy satisfaction, scullers, self‐determination theory, sports coaching

## Abstract

This study explored whether adult rowers' autonomy needs are differently met according to their age and how coaching status impacts on any differences. The numbers of older athletes motivated to engage in sport substantially lag behind their younger counterparts. Self‐determination theory suggests that motivation is underpinned by three universal, basic psychological needs: competence, relatedness and autonomy. Utilising a cross‐sectional design, 839 rowers aged 18–88 years old, predominantly from the United States of America, United Kingdom and other English‐speaking countries, were surveyed using the Psychological Need States in Sport Scale to assess how their autonomy needs were met. Two‐way ANOVA established that autonomy satisfaction (AS) increases, and autonomy frustration (AF) decreases as adult rowers age. Coaching was found to reduce AS and increase AF, but there was no significant interaction between age and coaching. This is the first study to establish that coaching both reduces AS and that older athletes have their autonomy needs better fulfiled than their younger adult counterparts. Conclusions draw on psychosocial theory to interpret why older athletes' autonomy needs may be less affected by exposure to autonomy‐frustrating environments. Current calls to make older athletes a special case by disproportionately providing more adult‐oriented coaching are challenged. Instead, it is posited that social factors, particularly higher tolerance of autonomy‐frustrating coaching styles for younger adult rowers, may mask ageism conventions that favour older athletes. Findings prompt recommendations for further research into how coaching interactions are constructed for athletes of different ages and their impact on AS and AF.

## Summary

- Older adult rowers report higher autonomy satisfaction and lower autonomy frustration compared to younger adults, suggesting autonomy needs become better met with age.
- Coaching reduces autonomy satisfaction and increases autonomy frustration regardless of age, challenging assumptions that coaching universally supports psychological needs.
- Findings call for coaching programmes to prioritise autonomy‐supportive practices for all ages, with potentially the greatest benefit for younger adults who appear more vulnerable to autonomy‐frustrating environments.

## 1 Introduction

The physical and mental health benefits of exercise for older adults are promoted internationally (Langhammer et al. 2018; World Health Organization [WHO], 2022). Despite recent increases (Hodge et al. 2008; Horton et al. 2019), the numbers of older athletes engaging in sport and physical activity substantially lag behind younger counterparts. The European Commission (2018) reports that 62% of 15–24‐year‐olds exercise or play sport, at least with some regularity, before steadily declining to just 30% of the 55+ cohort.

Understanding what sustains athletes' engagement through lifespan requires a closer examination of motivation itself. In sport and exercise psychology, motivation is generally considered to be a dynamic and multidimensional construct involving the internal and external forces that direct, energise and sustain behaviour (Roberts et al. 2007). Self‐determination theory (SDT) has established itself as one of the most validated and widely applied theories in contemporary motivation research, both in general psychology and in a sporting context (Ryan and Deci 2017, 2019; Standage and Ryan 2020). Deci and Ryan (1985) describe how satisfaction of three basic psychological needs (BPN) leads to more volitional and intrinsic motivation. BPN are considered essential for psychological growth, integrity and wellbeing: feeling effective and capable of achieving desired outcomes (competence), the sense of connection and belonging with others (relatedness) and the feeling of volition and sense that one's actions are self‐endorsed (autonomy). BPN can be satisfied or frustrated: for example, the concept of autonomy satisfaction (AS) is defined as ‘a feeling of psychological freedom and experience of integrity of one's actions, thoughts and feelings’ and autonomy frustration (AF) is the perception of ‘being controlled by external forces and internal conflicts’ (Deng et al. 2023, 4). In a sporting context, BPN thwarting might provide a potential explanation for reduced participation as adult athletes age.

Although SDT has been extensively applied to understand sports motivation, research is skewed towards younger athletes (Kim et al. 2019; Standage 2023). In contrast, a large proportion of adult athlete research focuses on Masters. ‘Masters athletes’ are generally individuals aged over 35, who are involved in organised competitive sports who have some form of enrolment or registration in a club or event and who acknowledge that they engage in some form of preparation to participate in sports (Young 2011; Young et al. 2014). Extant research assumes and confirms that Masters have different expectations to children, but relying on such a binary distinction overlooks whether there are nuanced needs across adult age groups (Dionigi et al. 2022). In addition, a large section of the wider adult athlete population remains under‐represented and less understood, that is, those aged between 18 and 34. This is a substantial omission.

Although SDT maintains that BPN are universal (Chen et al. 2015), how they are met for different ages can vary. Some studies report age‐related differences regarding the impact of cultural factors (Tang et al. 2021; Zhou et al. 2012). Further, Mackenzie et al. (2018) applied the SDT lens to examine how eudaimonic (meaning‐ and purpose‐based) well‐being changes across age groups. Eudaimonic well‐being is relevant as it focuses on self‐realisation, meaning in life, and psychological functioning beyond short‐term pleasure, making it particularly important in understanding long‐term motivation in sports (White and Yu 2019). However, studies into whether different aged adult athletes should be treated differently are limited (Young et al. 2018). This supports Teixeira et al. ’s (2012) assertion that the impact of demographics on motivation can only be understood by refining how SDT components are applied in exercise research.

Coaching is another recognised factor when considering whether athletes' BPN are likely to be met or not (Pulido et al. 2020). Research to date has mainly compared coaching styles. Sports coaches have traditionally been directive with emphasis on fault correction (Kerr et al. 2020). Directive coaching reduces the subject's sense of autonomy (Downey 2003). In contrast, more subject‐centred techniques have been shown to be effective in leadership and executive coaching domains (Loughead and Hardy 2005). This has prompted calls for a paradigm shift in sports coaching, to better align with SDT's humanistic foundation (Delrue et al. 2019; Occhino et al. 2014; Reynders et al. 2019). Adult‐oriented coaching directly applies SDT principles and includes athletes in decision‐making. Although terms such as ‘athlete‐centred’ and ‘adult oriented’ are open to individual and cultural interpretations (Cassidy 2010), the benefits of making adult athletes an active participant in coaching decisions and practice are well researched. This follows andragogic teaching principles, by presuming that adults respond well to having more autonomy, and fostering more equitable coaching relationships (Callary et al. 2017; Currie et al. 2021; Knowles et al. 2025).

Although adult‐oriented coaching has repeatedly been shown to improve all three basic psychological needs (Chu (Alan) and Zhang 2019; Currie et al. 2021; Motz et al. 2024), it is the autonomous element that moves the athlete from a passive coaching recipient to equal partner (Armold 2018). This assertion is particularly relevant to the aforementioned White and Yu (2019) study which found that BPN satisfaction increased with age, though the effect was most pronounced for autonomy. It suggests that ageing may be associated with a stronger alignment between values and behaviour, potentially influencing sustained sports engagement. Eagles and Callary (2023) demonstrate how this plays out practically, reporting that Masters athletes will capitalise on whatever autonomy they are afforded to ensure that other needs are met: In their study, higher performing athletes and those selecting coaches online gravitated towards adult‐oriented coaches. This indicates that autonomy may be critical to facilitating the other two BPN. Indeed, Bartholomew et al. (2009) specifically describe how autonomy supportive coach behaviours act as the catalyst to for relatedness and competence.

Adult‐oriented coaching is now frequently endorsed in coach training programmes (e.g., England Athletics, n.d.; Vinson and Bell 2020) and has even been adopted nationally (Special Olympics Coaching Fellowship 2013). Nevertheless, contradictions between theory and practice persist. Coaches are commonly recruited for their technical knowledge rather than their andragogical prowess (Chroni and Dieffenbach 2021). This need not be a barrier, as adult‐oriented coaching can be learnt (Belalcazar et al. 2024; Su and Reeve 2011). However, selecting coaches purely on technical merit results in coaches adopting their preferred style, with many merely replicating how they were taught (Camiré et al. 2012; Erickson et al. 2007). This conflict suggests that the benefits of contemporary coaching styles may not yet be sufficiently embedded for adult athletes' autonomy needs to be universally met. Hoffmann et al. ’s (2020) research lends support to this argument: They found that coaching as a general entity frustrates Masters athletes' sense of autonomy. This is important as Lataster et al. (2022) describe how AS generally increases with age. Tóth‐Király et al. (2018) balance this by reporting a slight reduction in AF among older adults, attributing it to them self‐selecting less autonomy‐frustrating environments. If it is accepted that older adults self‐select less autonomy‐frustrating environments, and as Hoffmann et al. suggest, the very action of being coached is autonomy frustrating, then this could provide some explanation as to why older adults participate less in sports. Further, it could be expected that adult athletes who expose themselves to coached sporting environments could expect increasingly lower levels of AS and higher levels of AF as they age. Despite the limited research into autonomy satisfaction and frustration changes across lifespan, any such age cohort differences in AS and AF for adults in sporting contexts remain largely untested. Moreover, Hoffmann et al.‘s findings were interesting but incongruent inasmuch as higher coaching frequency frustrated autonomy but an absence of coaching did not increase satisfaction. This makes it worthy of being partially replicated with another sample group drawn from the broader adult population and utilising an alternative survey.

Research into whether adult athletes' AS and AF change or remain consistent as they age is largely absent in extant literature. Findings that coaching increased AF but its absence did not increase AS for Masters athletes are anomalous and exclude 18–34‐year‐old adults. To the best of our knowledge, the paucity of research in these areas means that any interaction between adult athletes' coaching status, their age and the subsequent impact on meeting autonomy needs has never been studied. In light of these limitations, exploring the influence of age and coaching on the autonomy needs of adult athletes would advance knowledge and provide new perspectives in key areas.

### 1.1 The Present Study

This research sought to determine to what extent do age and coaching status affect the satisfaction or frustration of the BPN for autonomy in adult rowers, including potential interactions between these factors. It examined three hypotheses:

H 1 Adult rowers' basic psychological need for autonomy becomes increasingly frustrated as they age.

H 2 Adult rowers who receive more coaching are less likely to have their autonomy needs met.

H 3 There is an interaction between the coaching status of adult rowers and their age which impacts on whether their autonomy needs are met or frustrated.

## 2 Methods

### 2.1 Participants

For the purposes of this research, ‘rowers’ mean active, on‐the‐water rowers and scullers based on Sport England's (2023) definition of participation at least twice per 28‐day period. ‘Adult’ means any person who is 18 or over based on United Nations (n.d.) guidance. Although it is physically demanding and not accessible to all, rowing was chosen as it is a relatively low impact sport. This enables many athletes to continue to participate as they get older. High, international, masters level engagement can be observed in the world championships which often attract over 3000 competitors, with categories up to age 89+ (World Rowing, n.d.).

A priori analysis utilising G*Power version 3.1.9.7 software (Faul et al. 2007) for a nine cell, two‐way analysis of variables (ANOVA) was conducted to determine the minimum sample size required. To achieve 80% power, with a significance criterion of 0.05 and a medium effect size (*ƒ* = 0.25), a minimum sample size of 270 participants was required. A total of 839 participants (39% males, 60% females, 8 identified as nonbinary and 1 did not disclose, *M*_age_ = 53.69 years, SD = 15.70) provided their informed consent to take part in the research and fully completed the questionnaire. Of these participants, 45% resided in the USA, 30% in the UK, 8% in Canada, 7% in Australia, 5% in Ireland, 2% in New Zealand, 3% resided in other European countries and the Bahamas and 2 participants did not disclose their country of residence (see Table 1 for further demographic information).

**TABLE 1:** Age categorisation, participant numbers and demographics.

Adulthood age category	Early (18–41)	Mid (42–59)	Late (60+)	Totals
Overall participant numbers	178	346	315	839
Amount of experience				
< 2 years	54	11	43	108
2–5 years	50	24	58	132
> 5 years	74	311	214	599
Highest competitive level				
Hobby only	15	23	21	59
Club level	125	173	193	491
National/International	38	150	101	289
Whether participants are coached				
Yes	118	146	184	448
Sometimes	41	122	82	245
No	19	78	49	146
Whether participants coach others				
Yes	39	68	67	174
Sometimes	43	95	73	211
No	96	183	175	454
Gender				
Male	65	155	104	324
Female	106	190	210	506
Nonbinary	7	0	1	8
Prefer not to say	0	1	0	1
Adaptive				
Yes	18	10	15	43
No	160	336	300	796

*Note:* Adulthood age categories based on *A conception of adult development*, by Levinson 1986. Copyright 1986 by American Psychological Association.

### 2.2 Measures

#### 2.2.1 Demographic Information

Participants responded to questions about age, gender, country of residence and disability based on World Rowing's adaptive classifications. Information regarding the participants' coaching status was also collected. Hoffmann et al.’s (2020) survey question ‘Do you have a coach/instructor/cox that regularly supports you in your rowing/sculling?’ was utilised, although distinct functions, coaches, instructors and coxswains all provide guidance to athletes. This resulted in three categories: yes, sometimes, no. Three further questions established athletes' experience, competitive level and whether they also coach.

### 2.3 Psychological Need States in Sport Scale

Participants completed part of the Psychological Need States in Sport Scale (PNSSS) (Bhavsar et al. 2020). The 29‐question PNSSS measures basic psychological need satisfaction and frustration in autonomy, relatedness and competence. It was selected rather than Ng et al. ’s (2011) established Basic Needs Satisfaction in Sport Scale (BNSSS) which only measures basic psychological need satisfaction. The PNSSS better aligns with more contemporary SDT research by measuring both satisfaction and frustration in a single survey (Ryan and Deci 2017). It also allowed for the anomalous element of Hoffmann et al. ’s (2020) research into coaching status's impact on AS and AF to be partially retested using an alternative sample and survey.

As only autonomy was being assessed, autonomy‐relevant questions were extracted and used. This subscale consisted of a total of 10 questions measured along a 7‐point Likert scale, ranging from 1 (strongly disagree) to 7 (strongly agree). Questions 1, 3, 5, 7 and 9 assessed AS (*α* = 0.77), for example, Question 1 states ‘In rowing/sculling, I feel free to make choices with regards to the way I train’. Questions 2, 4, 6, 8 and 10 assessed AF (*α* = 0.81), for example, Question 10 states ‘In rowing/sculling, I must do what I am told’. Mean scores were calculated for both AS and AF, with a high score representing a high level of AS and AF, respectively.

### 2.4 Procedure

Following ethical approval, which was provided by the Ethics Panel at the [anonymised institution] on the 2nd October 2023, participants were recruited via social media and groups dedicated to on‐the‐water rowing communities. Data collection was conducted via a cross‐sectional design over five weeks (October to November 2023), where participants answered an 18‐question online survey, developed on Qualtrics (www.qualtrics.com).

### 2.5 Data Analysis

Several age groupings were considered, including World Rowing's 13 Masters categories that range from 27 to 89+. This would have excluded 18–27‐year‐olds, an important group and likely to receive more coaching than older counterparts. Moreover, World Rowing and many other sports and research categorisations are based on age‐related physical declination with associated psychological changes remaining implied. Instead, three categories based on Levinson's (1986) overlapping psychological life stages of early (18–41), mid (42–59) and late (60+) adulthood were utilised. This had two main advantages. First, age cohorts were formed according to established psychological theory, rather than an age‐related physical handicap system. Second, it amalgamated the very small numbers in some groups, particularly for the higher ages. This reduced cells for analysis from 42 to 9, thereby reducing the likelihood of Type I or II errors.

Data were analysed using SPSS 28.0. Two two‐way ANOVAs were conducted to evaluate the effects of age and coaching status on rowers'/scullers' AS and AF, respectively. Post hoc testing enabled a posteriori analysis and more specific conclusions to be drawn (Williams and Abdi 2010). Group mean comparisons incorporated a Bonferroni correction (Lee and Lee 2018) and partial eta squared was chosen to measure effect size rather than eta squared as it partials out other variable interactions (Richardson 2011).

## 3 Results

### 3.1 Data Screening

A total of 1084 responses were recorded on Qualtrics. About 245 responses were removed due to incompletion of the PNSSS, missing demographic information for the main analyses, such as age and coaching details, and having a low reCAPTCHA score (< 0.50). This resulted in a total sample size of 839 rowers/scullers (60% females, *M*_age_ = 53.69 years, SD = 15.70). Although Piovesana and Senior (2016) would advocate that the population size in this case was sufficient to assume no detrimental effect from skewed distribution, normality was tested. AS scores were negatively skewed (−1.33), and kurtosis was mesokurtic (*Z* = 2.10). Skewness for AF scores was 0.40, and kurtosis results indicated platykurtic distribution (*Z* = −0.68). All results were within acceptable bounds and normality assumptions were not breached (Byrne 2016). A Levene's test for equality of variance indicated that the homogeneity assumption was not breached for either AS (*F* = 1.67, *p* = 0.102) or AF (*F* = 1.31, *p* = 0.235).

### 3.2 Main Analysis

To assess the hypotheses, two two‐way ANOVAs were conducted to evaluate the effects of age and coaching status on rowers'/scullers' AS and AF, respectively. Table 2 presents the means and standard deviations for AS and AF, in relation to age and coaching status. Overall, on average, participants reported they felt their autonomy was fairly supported (*M* = 5.83), and that they experienced low AF (*M* = 2.92).

**TABLE 2:** Means and descriptive statistics for AS and AF in rowers and scullers.

Factor	Age categorisation	Coaching received	Mean (SD)
Autonomy satisfaction	Early adulthood (18–41 years old)	Yes	5.39 (0.98)
		Sometimes	5.33 (1.18)
		No	5.85 (1.06)
		Total	5.42 (1.05)
	Mid‐adulthood (42–59 years old)	Yes	5.68 (1.11)
		Sometimes	6.06 (0.92)
		No	6.00 (1.16)
		Total	5.83 (1.09)
	Late adulthood (60+ years old)	Yes	5.91 (0.99)
		Sometimes	5.92 (1.07)
		No	6.44 (0.95)
		Total	6.03 (1.03)
	All participants	Yes	5.68 (1.06)
		Sometimes	5.87 (1.07)
		No	6.21 (1.06)
		Total	5.83 (1.08)
Autonomy frustrated	Early adulthood (18–41 years old)	Yes	3.60 (1.27)
		Sometimes	3.65 (1.02)
		No	2.85 (1.49)
		Total	3.53 (1.27)
	Mid‐adulthood (42–59 years old)	Yes	3.17 (1.34)
		Sometimes	2.79 (1.22)
		No	2.62 (1.29)
		Total	2.98 (1.31)
	Late adulthood (60+ years old)	Yes	2.67 (1.31)
		Sometimes	2.68 (1.41)
		No	2.12 (1.27)
		Total	2.55 (1.35)
	All participants	Yes	3.12 (1.36)
		Sometimes	2.88 (1.34)
		No	2.38 (1.33)
		Total	2.92 (1.37)

Regarding H1, *adult rowers' basic psychological need for autonomy becomes increasingly frustrated as they age*, and a significant main effect was found for age on AS and AF, see Tables 3, 4, 5. Specifically, the mean AS score for early adulthood was significantly lower compared to mid‐adulthood (*p*_bonf_ = 0.005, *d* = │0.30│) and late adulthood (*p*_bonf_ < 0.001, *d* = │0.46│) and the difference between mid to late adulthood was not significant. The mean AF score for early adulthood was significantly higher than mid‐adulthood (*p*_bonf_ = 0.003, *d* = │0.38│) and late adulthood (*p*_bonf_ < 0.001, *d* = │0.70│). The mean AF score for mid‐adulthood was also significantly lower than late adulthood (*p*_bonf_ = 0.003, *d* = │0.26│).

**TABLE 3:** 2 × 2 ANOVA evaluating the effects of age and coaching status on rowers'/scullers' AS.

Source	Sum of squares	*df*	Mean square	*F*	*p*	$\eta_{\rho }^{2}$
Age	25.52	2	12.76	11.74	< 0.001	0.03
Coaching status	16.12	2	8.06	7.41	0.001	0.02
Age × coaching status	8.34	4	2.09	1.92	0.105	0.01
Error	902.59	830	1.09			

**TABLE 4:** 2 × 2 ANOVA evaluating the effects of age and coaching status on rowers'/scullers' AF.

Source	Sum of squares	*df*	Mean square	*F*	*p*	$\eta_{\rho }^{2}$
Age	64.49	2	32.25	18.88	< 0.001	0.04
Coaching status	31.82	2	15.91	9.32	< 0.001	0.02
Age × coaching status	7.12	4	1.78	1.04	0.384	0.01
Error	1417.35	830	1.71			

**TABLE 5:** Estimated marginal means for AS and AF.

Variable	Early adulthood	Mid‐adulthood	Late adulthood	Coached	Sometimes experience coaching	Not coached
Autonomy satisfaction	5.52	5.91	6.09	5.66	5.77	6.10
Autonomy frustration	3.37	2.86	2.49	3.15	3.04	2.53

Regarding H2, *adult rowers who receive more coaching are less likely to have their autonomy needs met*, and a significant main effect was also found for coaching status on AS and AF, see Tables 3, 4 to 5. Specifically, the mean AS score for those who experience coaching was significantly lower to those who do not experience coaching (*p*_bonf_ < 0.001, *d* = │0.38│), but not significantly different to those who sometimes experienced coaching. The mean AS score for individuals who are sometimes coached was also significantly lower than those who are not coached (*p*_bonf_ = 0.028, *d* = │0.28│). The mean AF score for those who experience coaching was significantly higher than those who do not experience coaching (*p*_bonf_ < 0.001, *d* = │0.47│). The mean AF score for individuals who are sometimes coached was also significantly higher than those who are not coached (*p*_bonf_ = 0.004, *d* = │0.34│). The difference between those who experience coaching and those who sometimes experience coaching was not significantly different.

Regarding H3, *there is an* *interaction between the amount of coaching adult rowers received and their age which impacts on whether their autonomy needs are met or frustrated*, and results revealed a nonsignificant age by coaching experience interaction for AS and AF. The differences in mean scores for both AS and AF for early adulthood rowers/scullers were not significant for individuals who experience coaching, sometimes experience coaching and those who do not experience any coaching. The differences in mean scores for both AS and AF for mid‐adulthood rowers/scullers were not significantly different for individuals who experience coaching, sometimes experience coaching and those who do not experience any coaching. The differences in mean scores for both AS and AF for late adulthood rowers/scullers were also not significantly different for individuals who experience coaching, sometimes experience coaching and those who do not experience any coaching. See Table 2 for the mean scores.

## 4 Discussion

This study aimed to establish whether adult rowers of different ages' autonomy needs are differently met and how coaching status impacts any differences. It set out three hypotheses to address these aims and inform the results. The results refute H1 by stating that adult rowers' psychological need for autonomy does not become increasingly frustrated as they age. The results do, however, support H2, suggesting that adult rowers who receive more coaching are less likely to have their autonomy needs met. As for H3, the analysis does not find a significant interaction between the adult rowers' coaching status and their age, which impacts whether their autonomy needs are met or frustrated.

This study found that adult rowers' basic psychological need for autonomy is better satisfied and becomes less frustrated the older they are. This relationship is significant but opposite to the prediction in H1. This finding is not entirely unexpected as it corresponds with more general AS and AF changes through lifespan. This finding adds to current knowledge in three main ways. First, it reinforces and consolidates the earlier cited studies. Specifically, it replicates Lataster et al. ’s (2022) findings of AS increasing with age, which is important as Tóth‐Király et al. (2018) found no such association. Second, it corroborates Tóth‐Király et al. ’s (2018) findings of reduced AF, which Lataster et al. (2022) never tested. Third, to our knowledge, this is the first study to establish a statistically significant relationship between AS, AF and age in an adult sporting context.

The analysis supports the H2 hypothesis that adult rowers who receive coaching are less likely to have their autonomy needs met than those who do not. These results corroborate and build on Hoffmann et al. ’s (2020) research. Findings add weight to their previous conclusions that coaching increases AF. It also confirms earlier findings that whether an athlete is coached to any degree makes the difference, and the amount has little additional impact. Arguably, the most important contribution of this study is the statistically significant finding that AS is higher when athletes are not coached. To our knowledge, this research is the first to establish that sports coaching has a negative impact on both AS and AF.

Seeking to mitigate the effects of AF in coaching programmes appears not be enough, and proactively incorporating more autonomy‐satisfying practice such as adult‐oriented coaching may be beneficial. Mageau and Vallerand (2003) distilled a number of key autonomy‐supportive behaviours that could inform practitioners and coaches to develop this approach. Their summary endorses providing choice, providing rationale, acknowledging athletes' feelings and perspectives, providing athletes with opportunities for taking initiative and independent work. They also include avoiding controlling behaviours such as overt control, criticisms, controlling statements, offering tangible rewards for interesting tasks and ego involvement in athletes.

Referring to H3, results suggest no significant interaction between coaching and age in relation to autonomy needs. This result was unexpected. If, as Tóth‐Király et al. (2018) found, older adults enjoy more autonomy by positioning themselves in environments that allow them to express their needs better, then exposure to the controlling style of sports coaching should work in opposition. The lack of interaction between age and coaching status is important in its own right as it identifies areas for future research. More specifically, it asks why adult athletes seem less affected by autonomy‐frustrating environments as they age. This study adds weight to the many calls for more autonomy‐satisfying coaching (e.g., Milbrath 2017; Reynders et al. 2019). It has practical implications for coaching programmes, as Su and Reeve's (2011) meta‐analysis found that autonomy‐satisfying instruction practice can be learnt. However, the results fundamentally contradict research calling for older people to receive disproportionately more adult‐oriented coaching due to different autonomy needs (Ferrari et al. 2017). Further exploration of the literature offers a potential explanation. It appears that coaching interventions are not equal for athletes of different ages. Contrasting coaching styles are often attributed to younger people being considered as representing the future and worthy of more intensive investment (Lyle 2020). However, this presumption risks overlooking the broader effects of age‐related psychosocial factors. Callary et al. (2015) offer critical insights. They describe Masters athletes' increased preparedness to repel autonomy‐frustrating coaching practice that younger athletes will more readily accept. This implies that Foucault's principles of unconscious and systemic power differentials may be at play (Foucault 2002). The degree to which someone experiences a sense of autonomy is inherently Foucauldian. Allain and Marshall (2017) summarise Foucauldian perspectives on ageing and exercise as an understanding of ‘the importance of micro‐relations in mobilising operations of power…and an attentiveness to the circulation of power and its resistance in everyday life’ (p. 404).

The apparent lack of interaction between age and coaching may be more of an indication of nuance requiring further research than a rebuttal of a relationship. MacLellan et al. (2017) and Motz et al. (2023) report how even the same coach will intuitively apply different coaching styles based on the athletes' age. This insight aligns well with Tóth‐Király et al.'s (2018) theory that older adults satisfy autonomy needs by either selecting suitable environments or modifying them to become autonomy‐satisfying. For older rowers, this may manifest in tacitly demanding a more adult‐oriented coaching style.

None of the above detracts from the benefits that older athletes would gain if this study's findings of AS reducing and AF increasing when athletes are coached were addressed in coach training. However, applying a psychosocial lens to the results suggests that considering older athletes as a special case to make sports more inclusive may be well‐intentioned but misguided. When describing ageism as the most socially acceptable prejudice, World Health Organization (2021) was clear that discrimination is experienced in both directions. Despite this, older generations form the basis of most ageism research (Burnes et al. 2019), with scarcely any directed towards early or mid‐adulthood (Kang and Kim 2022). If it is accepted that younger adults are more accepting of AF (Abrams 2010), then these results may represent the manifestation of prevailing power differentials and socially acceptable prejudice (West and Hewstone 2012). This would directly correspond with Tang et al. (2021)'s research into how culture impacts AS and AF for different age cohorts. The binary nature of researching junior to Masters athletes separately highlights differences that many would expect to observe. However, omitting 18–34‐year‐olds in adult studies overlooks the nuances of the transition and the broader effects of age‐related psychosocial factors that may be disadvantaging the young.

### 4.1 Applied Implications

Identifying that coaching interventions both increase AF and reduce AS has research implications by establishing congruence. This has practical implications by highlighting the hitherto, largely presumed need to increase AS for Masters athletes and not just reduce AF. It also demonstrates a need for researchers to consider including 18–34‐year‐old adults in future sport psychology studies. More importantly, by establishing that younger adults experience less AS and more AF than older cohorts, it challenges conventional wisdom on how coaches currently adapt their styles based on athletes' age (MacLellan et al. 2017; Motz et al. 2023). Although all ages would benefit from more adult‐oriented coaching, it implies that potentially bigger gains could be achieved by targeting younger adults to better meet their basic psychological needs.

### 4.2 Limitations and Recommendations

Participants were categorised by age and a simple self‐report of coaching status. With such a diverse population ranging from occasional clubs and hobby rowers through to elite international athletes, other factors might have introduced confounding variables. Examples include the time athletes commit to training, their previous experience, their personal goals and the proficiency and styles of their coaches. Measuring coaching's overall effect on autonomy satisfaction and frustration could overlook coaching style as an extraneous variable.

Despite broad geographical reach, this study only engaged English‐speaking participants and the moderating effect of culture between wider populations has not been fully considered (Schimmelpfennig et al. 2024). This is likely to be relevant as Zhou et al. (2012) found different reactions to teachers' autonomy‐frustrating behaviour between American and Chinese students. Such cultural differences in a teaching context are important and likely to similarly affect coaching relationships. Rowing coaching is more nuanced than many other team structures, with some boats incorporating coxswains performing a dual, crew member/pseudo coach role. All of the above impacts generalisability. Replicating this research, but adopting a qualitative or mixed methodology with participants from different cultures and other sports, would provide opportunity to incorporate more of these psychosocial factors. In order to test our interpretation of the results of this study, the qualitative element of the design should explore whether coaching encounters are constructed differently, dependent on age and how that impacts athletes' AS or AF.

## 5 Conclusions

This study is the first in a sporting context to confirm that older rowers experience increased autonomy satisfaction AS and decreased AF compared to younger counterparts and that any level of coaching reduces AS while increasing AF. Although these findings support earlier research, they also highlight a psychosocial paradox: despite inclusion efforts often focusing on older athletes, their autonomy needs are already better met, likely due to greater readiness to reject controlling coaching and elicit autonomy‐supportive interactions. In contrast, younger athletes may be more vulnerable to autonomy‐frustrating environments, challenging assumptions that they require less support. This reinforces SDT's claim that psychological needs are universal and emphasises the need for culturally and developmentally sensitive coaching practices across all age groups. By better understanding age‐related dynamics in autonomy‐need satisfaction, sports can move towards truly inclusive coaching that supports the health, motivation and well‐being of all athletes, not just older ones.

## Conflicts of Interest

The authors declare no conflicts of interest.

## Data Availability

The authors have nothing to report.
